# Transactions of the Pathological Society of Philadelphia

**Published:** 1840-03-21

**Authors:** 


### MEDICAL EXAMINER.

DEVOTED TO MEDICINE, SURGERY, AND THE COLLATERAL SCIENCES.

No. 12.] PHILADELPHIA, SATURDAY, MARCH 21, 1840. [Vol. III.

### TRANSACTIONS OF THE PATHOLOGICAL SOCIETY OF PHILADELPHIA.

*March*, 1840.

The President, Dr. Gerhard, in the Chair.

*Case of Dr. Parrish.* Reported by George B. Wood, M.D., *Professor of Materia Medica in the University of Pennsylvania.*

The following account of the case of the late Dr. Joseph Parrish is drawn up from memory, no notes having been taken during its progress.

When about twenty-five years of age, the Doctor was affected with a severe and lasting cough, and considered himself in danger of pulmonary consumption, to which he believed that he had a family predisposition, having lost a brother and sister by that complaint. Under a course, however, of vigorous exercise and free exposure to the air, without the use of medicines, he ultimately surmounted the threatening symptoms. The existence of cicatrices in the upper portions of the lungs, discovered upon post mortem examination, proves that his apprehensions were well founded, and at the same time affords strong evidence in favour of the plan of treatment which he adopted in his own case, and always strenuously advocated.

For many years after the disappearance of the pectoral affection, Dr. Parrish suffered much from dyspepsia, which continued with him even till middle life. But from this also he was at length relieved, and afterwards enjoyed good health, with the exception of occasional severe attacks of catarrh during the winter, and of irregular gouty or rheumatic affections, particularly of lumbago, which usually continued but for a short period. In one of these attacks, however, of irregular gout, which occurred about eleven years previously to his last illness, he was confined to his bed a long time, and suffered extremely. The disease appeared to affect chiefly his stomach, diaphragm, intercostal muscles, and upper extremities; and he was at one time considered in imminent danger. He had a slow convalescence, but recovered entirely, without any apparent relics of the disease, which could give rise to uneasiness. His predisposition to gout was thought to be hereditary.

It may be proper to mention, in reference to the state of the kidneys and prostate glands, revealed by dissection, that he laboured, during the greater part of his life, under more or less irritation of the urinary passages, and that the secretion of urine was more copious than is usual in health. He believed that in early life he had suffered from a slight attack of diabetes.

These preliminary facts are stated, as they may throw some light upon the character of the affection of which he died, and upon the phenomena observed after death.

The health of Dr. Parrish began to decline in the early part of last summer. He lost flesh, and felt uncomfortable, without being able to ascribe his condition to any particular cause. In August, 1839, he visited Saratoga, with the view of improving his health, and while absent from home, was attacked with a severe neuralgic affection of his lower extremities. This was so far relieved by opiates as to enable him to return to Philadelphia, but did not entirely disappear for several weeks. The chief seat of the affection was in the calf of the left leg. The pain was excruciating, but without heat, redness, or swelling of the part. It yielded to very copious local depletion, by means of leeches.

After this attack he never recovered his former health. His appetite was impaired, his bowels often costive, his sleep disturbed and not refreshing; and, though he continued to attend to his professional duties, he exhibited less energy than formerly, and in the afternoons was indisposed to exertion, sleeping much in his chair, and often waking up in a debilitating perspiration. Soon after the commencement of winter, he was seized with catarrh, which gradually increased, and at length became so severe as to render confinement prudent if not necessary. It was not, however, till about the first of January that he concluded to remain at home and try the effect of remedies.

He was now much more emaciated, had a pulse of about one hundred, and apprehended phthisis; and it was the conviction that he was predispossd to this complaint which had induced him to delay confinement. But the existing pectoral symptoms were obviously those of bronchial inflammation, and yielded very considerably to a slight mercurial impression produced by the blue pill. The general amelioration of his condition, under the influence of mercury, was indeed very striking. Expectoration, which was before deficient, became established, the cough was diminished in violence, and the pulse sunk down to the natural standard. He was so much improved as to be able to leave his house occasionally, and even to visit patients, when the weather was favourable. But he was still far from being well. The emaciation continued, and the cough, though moderated, did not altogether leave him, but took on a laryngeal character, as if the irritation which produced it was seated chiefly in the glottis. The pulse again became more frequent than natural, and he began to have a sallow hue, though the alvine evacuations and the urine were little, if at all, changed in colour. Petechiæ made their appearance pretty thickly upon the lower extremities, but were not permanent. Though the appetite was somewhat impaired, enough food was taken for healthy nutrition, had not some latent cause existed to disturb the process.

After exposure out of doors, and fatiguing professional exertion, on the 29th of February he became so much worse as to be confined to his room, which he did not afterwards leave. His cough was now almost exclusively laryngeal, and occurred paroxysmally towards the close of the day, when it became exceedingly violent, and continued almost incessantly for hours. The sensation in his throat was such that he could scarcely convince himself that there was not a foreign body in the larynx. The slightest attempt to speak brought on violent convulsive attacks of coughing, which were attended with shooting pain in the throat, and not unfrequently also with vomiting, consequent, probably, upon the agitation of the parts about the fauces. The stomach was at length thrown into a state of sympathetic irritation, which continued even during the absence of the cough, so that scarcely anything swallowed could be retained. The pulse was now usually between ninety and one hundred, soft and moderately full; the skin soft and of a natural temperature; the tongue slightly furred and disposed to dryness; the secretion from the kidneys and liver very nearly healthy in appearance, though the evacuations from the bowels were sometimes rather too light coloured, and the urine rather too yellow. The sallowness and emaciation had somewhat increased, and a slight disposition to lethargy was observable, which appeared to have existed for some time previously, though it had not attracted particular attention. There was no unusual irritation of the urinary passages; no pain in the small of the back, nor in the right side or shoulder; no difficulty in lying in any position; very little, if any, tenderness in the right hypochondrium, in short, none of the usual evidences of inflammation of the kidneys or liver, though the lethargic symptoms, and the somewhat jaundiced condition of the skin and eyes, might seem to point to these organs as the seat of disease.

A blister was applied to the throat; the blue mass and hyoscyamus were taken in small doses frequently repeated; cream or milk, with lime water, was employed as food, and to allay the sickness of stomach. The gums soon became somewhat sore, without any material amendment of the symptoms, and the mercurial was omitted. An attempt was made to interrupt the paroxysms of coughing, by quinia; but this was omitted, after a few grains had been taken, in consequence of the irritation of stomach which it produced. With the same view, enemata of laudanum were administered in anticipation of the paroxysm for two or three days successively. This remedy produced the desired effect, and the harassing laryngeal cough ceased almost entirely, but the irritation appeared merely to have receded, and the increase of the lethargic symptoms, and the almost constant sleepiness which came on, indicated, but too plainly, that it had been transferred to the membranes of the brain.

Up to this period, Dr. Parrish, though occasionally seen by medical friends, had kept the management of the case in his own hands. Drs. Wood and Otto were now called into consultation with Dr. Isaac Parrish; though even till within two days of the close of the disease, the mind of the patient, when sufficiently aroused, acted with entire clearness, and his opinions were necessarily allowed some weight in the treatment. Towards the close of the case, Dr. Hewson was associated in the consultation.

A few leeches were applied to the temples, and mustard to the feet. Calomel was given in minute and frequently repeated doses, and the diet was continued as before. The drowsiness was somewhat diminished, and the stomach became more retentive, but the case was complicated by the occurrence of singultus, which gradually increased, and soon became one of the most prominent symptoms, continuing, with occasional interruptions, till a day or two before death. Neuralgic pains also occurred in the left thigh and leg, and some soreness was observed along the upper part of the spine. The latter affection increased by degrees, occupying chiefly the back of the neck and the left shoulder, and became at length so severe as greatly to impede motion. Towards the close of the case, it constituted almost the sole source of complaint.

The remedies employed were chiefly calomel in small doses, as before; musk, creosote, assafetida by enema, and other antispasmodics, for the relief of the hiccough; magnesia and rhubarb, with terebinthinate enemata, as laxatives; blisters to the extremities, and over the stomach; acetate of morphia in small quantities to the blistered surface in the epigastrium, and dry cups to the back of the neck, with the abstraction of an ounce or two of blood from the same part. The diet consisted chiefly of cream and chicken broth. There was a constant desire for cold drinks, in which the patient was indulged, carbonic acid water being preferred, as it quieted the irritability of the stomach. Opiates, moderately given, relieved distress without increasing stupor, but were thought to be followed, when their immediate effects went off, by an increase of nausea, and were used as little as possible. The loss of blood, though indicated by some of the local symptoms, was forbidden by the steadily increasing prostration, and even the slight local bleeding was not well borne.

The treatment seemed to have no other effect than a temporary alleviation of the distressing symptoms. The disease marched steadily onward. The lethargy increased, the skin became quite jaundiced, the abdomen tympanitic, the pulse more frequent and feeble, and the bowels obstinately costive. Subsultus tendinum came on, but there was no paralysis. The only additional remedies employed were the oil of turpentine, and aromatic ammoniated alcohol. Towards the end of the complaint the sight and hearing were greatly impaired, and for twenty-four hours before death seem to have been altogether lost. In the midst of the lethargy there were evidences of considerable physical uneasiness, but this entirely disappeared during the last three or four hours, and life was gradually extinguished without convulsions, or other distressing symptoms.

Death occurred on the 18th of March.

*Notes of an examination of the body of Dr. Joseph Parrish,* March 19, 1840, thirty-two hours after death. Present Drs. Otto, Wood, Ashmead, Pennock, Gerhard, Kirkbride, Barton, and West.

Jaundiced tint of skin—Emaciation of face very decided,—no infiltration.

*Head.*—Scalp unusually dry and bloodless; the yellowness extending to the pericranium: adhesion of dura mater to cranium unusually strong; veins of upper part of brain nearly empty; at least three ounces of serum exuded from beneath the arachnoid; this membrane upon its upper surface *semi-opaque*, and elevated by the effused serum; arachnoid not adherent, cloudy, not granulated, quite pale; convolutions distinct, pale, firm; consistence of upper part of cerebrum even greater than usual; both substances extremely pale, with a faint, yellowish tinge; cortical substance projecting above the level of the medullary, and rather disproportionately firm; cerebral substance, in general, extremely firm. One or two drachms of serum in the ventricles. *Plexus Choroides* moderately injected. *Thalami* and *corpora striata* firm and normal.

*Base of brain* offered about two ounces of serum; appearance of this part remarkably healthy, with barely average injection. The membranes are scarcely injected or opaque; substance of brain and cerebellum pale and firm.

*Thorax.*—On right side some adhesions are found posteriorly, with effusion into pleura of half a pint of reddish serum. The right lung at its summit presents an irregular puckered depression, which is firm and opaque; upon cutting into it, there is found a substance for the most part of a dirty grayish colour, nearly cartilaginous in consistence, with some grains of a calcareous matter in it, evidently the cicatrix of an old tubercular cavity of small size. From the back part of this depression there extends another one, which is more irregular, running round the lung, and connected with similar deposit. Upon stretching and pulling the lung slightly, cellular tissue may be discovered passing through this hardened mass, and apparently constituting it. Several large vessels may be seen passing around the above cellular substance; one or two slighter depressions of the same kind are found in the adjacent parts of the lung. The substance of the lung generally is gorged with serum, crepitous, and contains air; the vessels are more or less enlarged; the pleura of the upper third of the lung is more or less opaque, giving to the whole of this portion of the lung a peculiar wrinkled aspect, less marked, however, than at the summit. The mucous membrane of the bronchial tubes, opaque, jaundiced, but not reddened.

*Left lung.*—Nearly as much serum in the pleural cavity of this side as in the right one, of same character; the lung free from adhesions; its lower lobe more engorged with blood than the right, but containing less serum; still crepitous throughout; upper lobe in its lower three-fourths paler than natural; some emphysema along its anterior margin; tissue engorged with bloody serum; containing air; its upper one-fourth hardened and puckered, but less so than on the right side; whole tissue somewhat solid and condensed. Several masses of cellular substance of the same colour as those in the right lung are found in it, two of which are particularly well defined, and present an irregular central nucleus, one-eighth of an inch in diameter, from which run radii, of firm cellular substance; appearances still more characteristic of cicatrization. No *recent* tubercular granulations, nor any trace of recent tubercular deposition are any where discoverable.

*Heart.*—The pericardium contains about two ounces of bloody serum; the surface of heart is remarkably free from opacity; no adhesion of pericardium, except slightly at the vessels; left ventricle soft, slightly jaundiced; a little thickening about the mitral valve. Semi-lunar valves flexible, but a little cartilaginous at their base, not, however, sufficiently to interfere with their action. Some cartilaginous deposit beneath the inner membrane of the aorta immediately above the valves. Right ventricle of the same colour with the left; stained somewhat yellowish; valves flexible.

*Abdomen.*—The peritoneum contains more than a quart of reddish-yellow serum. The *liver* is extremely irregular in outward appearance; its surface is completely studded with irregularly rounded elevations. Some of these are about the size of grains of rice, others two or three times as large. The left lobe is shrunken to half its size; the right a little less than usual. The tissue of the liver is extremely heavy and condensed. The tuberosities of its substance are more marked in the left than in the right lobe, and vary in size from a pin's head to a hazle-nut. The colour is a light fawn—cellular substance much atrophied. The seat of the morbid deposit is evidently in the acini, which vary extremely in size. The blood in the vessels of the liver is thin and watery. The left lobe, when incised, is paler than the right one, and the extreme cartilaginous firmness is even better marked in it. The granulations of the substance are also much more distinct and numerous in the left lobe. The liver is *not fatty*, but on the whole presents a remarkable specimen of that peculiar change in its structure termed cyrrhosis. The bile is thin and greenish; in the gall bladder are found *six* dark green mulberry calculi, about the size of peas—the lining membrane of this part is thin and pale.

*Spleen.*—Rather firmer than natural; six inches by four.

*Stomach.*—Of a greenish and reddish colour at its great cul-de-sac, evidently from commencing decomposition; it contains a little thin mucus; its cardiac extremity is of a deep red colour, chiefly from imbibition; its lining membrane has been finely injected—not softened; rest of its surface marbled with red and green, mamellated and slightly thickened. (*Note.*—The commencing state of decomposition of the stomach makes these lesions rather doubtful, but evidently this organ has been the seat of chronic inflammation and thickening.)

*Kidneys.*—Left one very large, with great abundance of fat about it; upon removing it, we opened into a sac which was filled with purulent liquid; the sac containing the pus occupies half the anterior, and the whole posterior of the kidney, except at its summit—it is formed by the proper membrane of the kidney, which is detached from it, and in this way constitutes the abscess. The posterior part of the kidney presents, near its inferior extremity, an adhesion an inch and a half in diameter, rounded, the membrane adjoining it is evidently a purulent membrane, which here and there has small, rounded ulcerations in it. The yellow spots, observable on its surface, connected with the membrane of the kidney, and forming the points of adhesion to it, constitute the exterior of the masses of purulent infiltration into the cortical substance of the kidney. The cellular substance of the kidney remains, but no trace is to be found of the true renal structure: near, and above this, is another purulent infiltration of irregular size, of which there are several throughout the cortical portion, constituting, in fact, small abscesses, the largest of which will contain an almond. About one-fourth of the kidney retains, in part, its normal appearance, but is filled with the yellow granulations of the acini, which are evidently those of Bright's disease. The exterior of this portion of the kidney presents a yellowish chagrined appearance from the projection of the same granulations. The whole of the kidney is enlarged about one-third, lobulated; the ground work of it is of a more livid colour than usual, and soft.

*Right kidney* about of natural size; investing membrane adheres closely to it; on separating it, the same yellow points, mentioned before, were observed, some of which are from one-half to three-quarters of an inch broad, prominent, soft, and, on penetrating them, they are found to be filled with pus of perfectly healthy appearance. The substance of the kidney, generally, is of a deep red colour, dotted with darker brownish points; at the posterior and inferior part, on detaching the membrane, we open into an abscess containing about two ounces of perfectly creamy, laudable pus, which is contained in a similar infiltrated mass, formed by the cellular substance, here and there thrown into *poches*, (very similar to forming abscesses in the lungs,) which do not seem to communicate with the calices in either kidney. The rest of the kidney, in its cortical substance, is filled with minute granulations, less marked, however, than in the left one; the cortical substance is soft, reddish, and evidently inflamed.

*Bladder* very nearly natural; slight roughness of its lining membrane, at its inferior portion, like grains of sand on it.

*Pancreas* contains more blood than usual; harder; ecchymosed throughout; lobules firmer than usual, harder.

*Prostate gland* not enlarged, its texture changed internally in about one-half of it, to an almost cartilaginous firmness, of a yellowish tinge, not grating under the knife; upon squeezing it, a purulent fluid is poured out, apparently the secretion of the glands; upon cutting into it, the same fluid is seen in small points through it.

*Large intestines* opened in several places; contain yellow fœces; mucous membrane of a slate colour; no ulceration found at any point observed.

*Small intestines* of same appearance, apparently perfectly healthy.

---

At a meeting of the Pathological Society of Philadelphia, held March, 1840, the following resolutions were adopted:

Resolved, That the Pathological Society have learned with deep regret the demise of their respected associate member, Dr. Joseph Parrish, whose philanthropy and devotion to the science of medicine, have for so many years commanded the high respect and regard of the members of his profession, and will ever be remembered by them with the liveliest gratitude.

Resolved, That the Society highly appreciate the importance of the directions given by Dr. Parrish, that his body should be examined, believing that the example is one which is eminently conducive to the advancement of the science of medicine, and to the deepest interests of humanity.

GEO. W. NORRIS, *Secretary.*

At a special meeting of the Philadelphia Medical Society, held on the 19th of March, 1840, the Vice President, Professor SAMUEL JACKSON, being in the Chair, the following preamble and resolutions were adopted :

Whereas, It appears to be proper to this Society that, upon the decease of an eminent member of this body, some expression of the sentiments of his remaining brethren upon the occasion should be made public, and,

Whereas, It hath pleased the Divine Providence to remove from this world our late esteemed and beloved member, JOSEPH PARRISH, M. D., it is, therefore,

Resolved, That we have heard with profound regret of the decease of that eminent physician, whose professional skill, humanity, and liberal and just conduct, have long endeared him to us, and to the profession at large in this city.

Resolved, That we deplore the loss of a physician whose long career in the practice of his art has been distinguished by the most brilliant success, accompanied by a modesty and ingenuousness of conduct, which secured to him the affectionate attachment of the medical profession in this city, as well as their highest respect.

Resolved, That a member of this Society be requested to prepare and deliver before this body, a biographical account of the deceased, in order that, although his present and living example has ceased from among us, some permanent memorials may be secured, of a life which has been highly useful to the medical body, by a pure example of morals, by great attainments in medicine and surgery, and by a rare moderation, fit to be preserved as the model of a wise, benevolent, and upright physician.

Resolved, That the members of the Society wear crape on the left arm for thirty days, as a testimony of their feelings, as expressed in the foregoing resolutions.

Resolved, That the members of the Society will attend the funeral of the deceased, to-morrow afternoon, at three o'clock, and that we hereby invite the physicians of this city to meet with us at our Hall, in order to join with us in the manifestation of due respect to the deceased.

Resolved, That a committee of three members be appointed to present a copy of these resolutions to the family of the deceased.

Resolved, That Dr. GEORGE B. WOOD be respectfully requested to prepare the memoir contemplated in the above resolutions.

Resolved, That an account of the proceedings of this meeting be published in the medical periodicals of this city, and in the city morning papers of to-morrow.

EDWARD HARTSHORNE, Jr., *Rec. Sec.*
*Philadelphia*, March 19, 1840.

At a meeting of the former pupils of the late Dr. JOSEPH PARRISH, held at the hall of the Medical Society on the 21st of March, 1840, Professor WOOD was called to the Chair, and Dr. NORRIS appointed Secretary. The following resolutions, offered by Prof. MORTON, were adopted :

Resolved, That we contemplate with feelings of unfeigned sorrow, the demise of our venerated friend and preceptor, JOSEPH PARRISH, M. D.

Resolved, That we regard the death of this estimable man as a severe loss, not only to the medical profession, but to the community at large, and above all, to ourselves, who have been instructed by his precepts, and fostered by his kindness.

Resolved, That an intimacy of many years continuance, has tended more and more to enhance our esteem for his many virtues, his amiable manners, and his professional skill.

Resolved, That a copy of these resolutions be presented to the family of Dr. Parrish, with the assurance of our sincere sympathy for the afflictive bereavement they have suffered in his death.

Prof. MORTON and Dr. YARDLEY were then appointed a committee to carry into effect the last resolution.

On motion of Dr. YARDLEY, it was

Resolved, That this meeting cordially unite with the Medical Society in the appointment of Professor WOOD (one of our number) to prepare a biographical memoir of our lamented preceptor; believing that he will do full justice to his superior ability as a teacher of medicine and surgery, as well as pourtray his exalted and endearing character as a physician, his worth as a man, and his virtues as a Christian.

Resolved, That we will in a body attend the reading of said memoir, at such time and place as Dr. WOOD and the Medical Society may select.

On motion of Dr. WEST,

Resolved, That the proceedings of this meeting be published in the medical periodicals and newspapers of the city.

GEO. W. NORRIS, *Secretary.*

